# Deep gluteal syndrome

**DOI:** 10.1093/jhps/hnv029

**Published:** 2015-06-06

**Authors:** Hal David Martin, Manoj Reddy, Juan Gómez-Hoyos

**Affiliations:** 1. Hip Preservation Center at Baylor University Medical Center at Dallas, 3900 Junius St. Suite 705, Dallas, TX 75246, USA,; 2. Texas A&M Health Science Center College of Medicine at Dallas, 3950 N A.W. Grimes Blvd, Round Rock, TX 78665, USA and; 3. Department of Orthopaedic Surgery, University of Antioquia, Medellin, Colombia

## Abstract

Deep gluteal syndrome describes the presence of pain in the buttock caused from non-discogenic and extrapelvic entrapment of the sciatic nerve. Several structures can be involved in sciatic nerve entrapment within the gluteal space. A comprehensive history and physical examination can orientate the specific site where the sciatic nerve is entrapped, as well as several radiological signs that support the suspected diagnosis. Failure to identify the cause of pain in a timely manner can increase pain perception, and affect mental control, patient hope and consequently quality of life. This review presents a comprehensive approach to the patient with deep gluteal syndrome in order to improve the understanding of posterior hip anatomy, nerve kinematics, clinical manifestations, imaging findings, differential diagnosis and treatment considerations.

## INTRODUCTION

The progress in understanding posterior hip anatomy and sciatic nerve kinematics has helped to identify several locations where the sciatic nerve can be entrapped. For that reason the term ‘deep gluteal syndrome’ instead of ‘piriformis syndrome’ is now preferred to describe the presence of pain in the buttock caused from non-discogenic and extrapelvic entrapment of the sciatic nerve [1]. The structures that can be involved in sciatic nerve entrapment within gluteal space include the piriformis muscle [2, 3], fibrous bands containing blood vessels, gluteal muscles [4], hamstring muscles [5, 6], the gemelli-obturator internus complex [7, 8], vascular abnormalities [9, 10] and space-occupying lesions [11].

There are some unique factors in the history and physical examination that can define the specific site where the sciatic nerve is entrapped. A comprehensive assessment of the hip will help to recognize the specific problem in each individual case. Failure to identify the cause of pain in a timely manner can increase pain perception, and affect mental control, patient hope and consequently quality of life. This review presents a comprehensive approach to the patient with deep gluteal syndrome in order to improve the understanding of posterior hip anatomy, nerve kinematics, clinical manifestations and treatment considerations as well as the differential diagnoses like pudendal nerve entrapment, ischiofemoral impingement, greater trochanter ischial impingement and ischial tunnel syndrome.

## DEEP GLUTEAL SPACE DEFINITION AND SCIATIC NERVE KINEMATICS

**Fig. 1.**
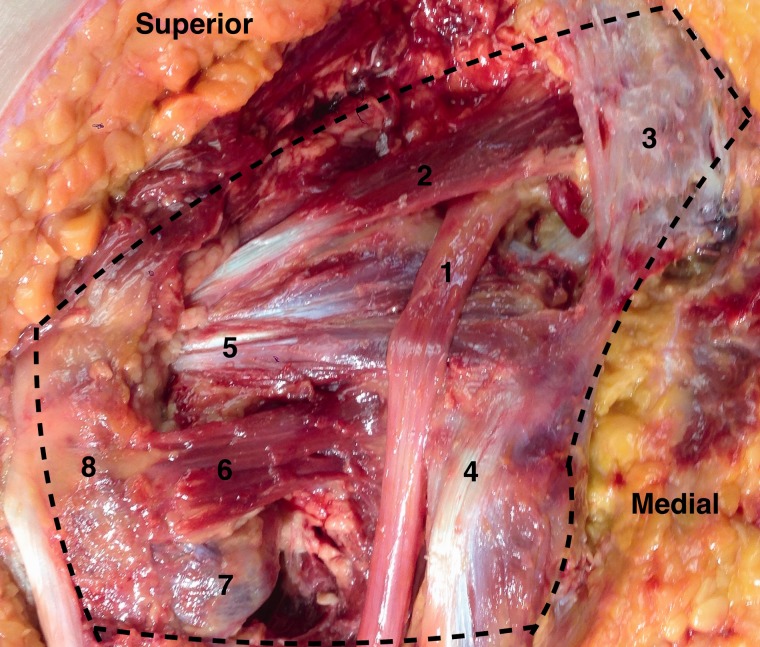
Deep gluteal space. A cadaveric left hip with the gluteus maximus reflected. The course of the sciatic nerve (1) as it enters the pelvis at the sciatic notch anterior to the piriformis muscle (2) and sacrotuberous ligament (3). As the nerve courses distally toward the ischium and hamstring origin (4) it passes posterior to the gemelli-obturator internus complex (5) and quadratrus femoris (6, with the inferior portion removed to expose the lesser trochanter). Lateral structures include the lesser trochanter (7) and greater trochanter (8).

The borders of the deep gluteal space are defined by the following: (i) Posteriorly, the gluteus maximus, (ii) Anteriorly, posterior acetabular column, hip joint capsule and proximal femur, (iii) Laterally, lateral lip of linea aspera and gluteal tuberosity, (iv) Medially, sacrotuberous ligament and falciform fascia, (v) Superiorly, inferior margin of the sciatic notch, (vi) Inferiorly, proximal origin of the hamstrings at ischial tuberosity (Fig. 1) [4].

The anatomy of the deep gluteal space is unique in that the nerve enter the pelvis through the sciatic notch and it has a significant mobility with hip movements as described by Coppieters *et al*. [12]. They found that the sciatic nerve has 28 mm of excursion during hip flexion. However, this motion is affected by anatomic variances between the sciatic nerve and the piriformis muscle in 16.2% of the population as reported by Smoll *et al*. [13].

Considering that a neuropraxia can be produced at 6% strain, and that a complete block can occur at 12% strain [14], the nerve kinematics is a crucial aspect of the entrapment’s pathophysiology. To date, the kinematics of the sciatic nerve continues to be explored. It has been reported that in deep flexion, abduction and external rotation of the hip, the sciatic nerve glide across the posterior border of the greater trochanter. Additionally, in the full flexed, abducted externally rotated state, the semimembranosus origin and the posterior edge of the greater trochanter can come into contact [15]. Excursion of the nerve is dependent upon the flexion of the knee. When the knee is flexed, the nerve moves posterolateral and when the knee is extended the nerve moves deep into the tunnel [15].

### History

The diagnosis of deep gluteal syndrome is based upon a comprehensive history and physical examination of the hip (see video on Youtube® https://www.youtube.com/watch?v=IhvVoKGyl8E).

The lumbar spine plays an important role in hip pain. Hence, a differentiation of problems coming from the spine should be performed during the history and physical examination. Once the lumbar pathology is ruled out using specific tests and spine imaging, the physical examination should be directed toward the deep gluteal space as a cause of posterior pain.

Patients with sciatic nerve entrapment often have previous history of trauma and symptoms of sit pain, radicular pain of the lower back or hip and paresthesias of the affected leg [4, 16]. Patients may present with neurological symptoms of abnormal reflexes or motor weakness [17]. Some symptoms may mimic a hamstring tear or intra-articular hip pathology such as aching, burning sensation or cramping in the buttock or posterior thigh.

The factors or positions that increase or decrease the pain can dictate the diagnostic approach. A sitting pain is usually associated with sciatic entrapment beneath the piriformis muscle. A walking pain lateral to the ischium is associated with ischiofemoral impingement, in which the lesser trochanter rubs the lateral border of the ischium [18].

Filler *et al*. [3] originally described the sources of non-discogenic sciatic nerve entrapment in 239 patients, and the most common sites were beneath the piriformis muscle (67.8%), sciatic foramen (6%), ischial tunnel (4.7%), followed by the additional sources as seen in Table I.

**Table I.** Diagnostics established between 239 patients with sciatica [3].

Diagnosis	Percentage of patients
Piriformis syndrome	67.8
Distal foraminal entrapment	6.0
Ischial tunnel entrapment	4.7
No diagnosis	4.2
Discogenic pain w/ referred leg pain	3.4
Pudendal nerve/Sacrospinous ligament	3.0
Distal sciatic entrapment	2.1
Sciatic tumor	1.7
Lumbosacral plexus entrapment	1.3
Unappreciated lateral disc herniation	1.3
Nerve root injury due to spinal op	1.3
Inadequate spinal root decompression	0.8
Lumbar stenosis presenting as sciatica	0.8
Sacroiliac joint inflammation	0.8
Sacral fracture	0.4
Tumor in lumbosacral plexus	0.4

See original article by Filler *et al*. for the diagnostic process.

Posterior hip pain could originate from intrapelvic problems, hence a focused urologic and gynecologic history should be addressed in order to detect any suggestive sign of intrapelvic entrapment such as cyclic variations in pain [19]. High resolution imaging of the pelvis or abdomen assists in identifying any type of endometrioma or vascular entrapment of the sciatic nerve in the intra-abdominal region.

Posterior hip pain commonly presents in a chronic fashion significantly affecting the quality of life. Therefore, a psychological evaluation before any treatment is recommended for patients complaining of chronic pain >6 months, who are using narcotics, and have a significantly impaired social function. Psychological strategies for diagnosing depression and anxiety disorders related with chronic pain have shown high specificity and reliability [20]. These factors could affect the outcomes and the satisfaction of the patient and the surgeon.

### Specific tests on physical examination

A five-level examination is mandatory in the diagnosis of complex disorders of the hip. The examination will adequately assess the osseous (level 1), capsulolabral (level 2), musculotendinous (level 3), neurovascular (level 4) and kinematic chain (level 5) helping to direct appropriate tests and treatment modalities [21].

A common language and technique on physical examination should be used [22]. Patients presenting with chronic pain should have a systematic exam to ensure that important causes of posterior hip pain are not overlooked.

**Fig. 2.**
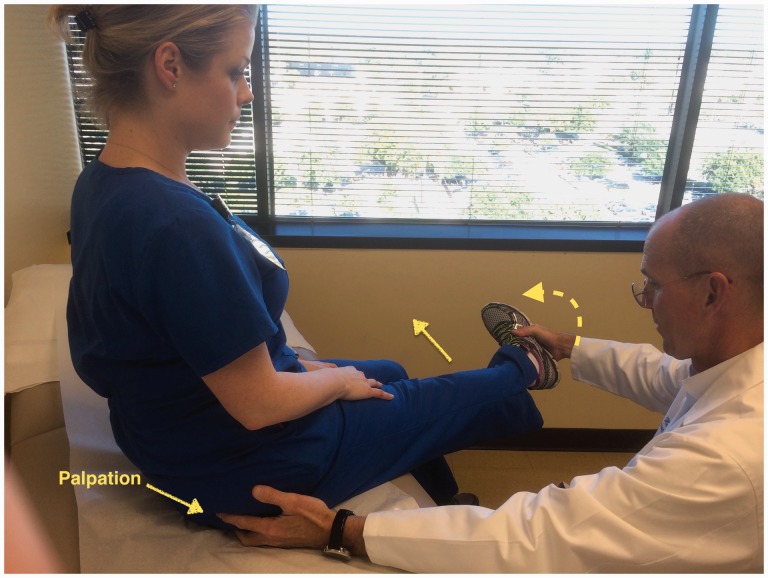
Seated piriformis stretch test. The patient is in the seated position, which offers a stable reproducible platform with 90 degrees of hip flexion. The examiner extends the knee (engaging the sciatic nerve) and passively moves the flexed hip into adduction (solid arrow) with internal rotation (dashed arrow) while palpating 1 cm lateral to the ischium (middle finger) and proximally at the sciatic notch (index finger). A positive test is the recreation of the posterior pain at the level of the piriformis or external rotators.

**Fig. 3.**
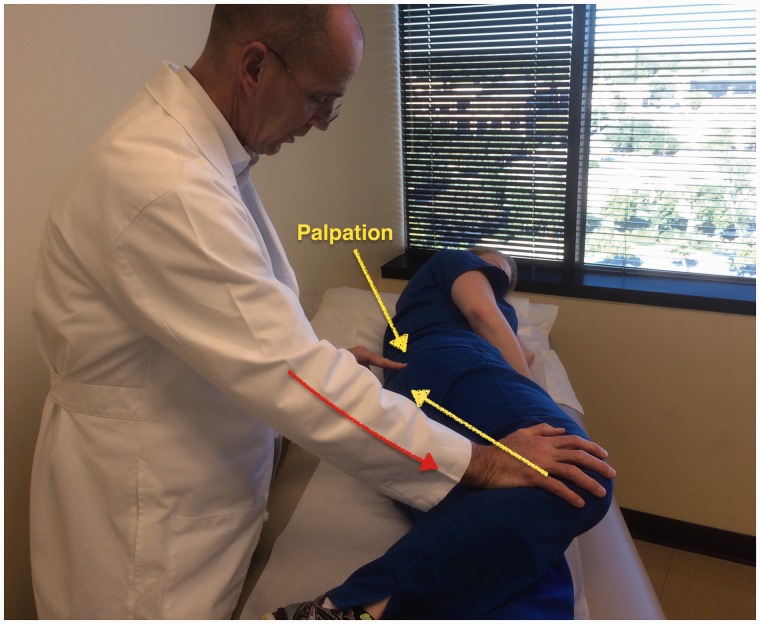
Active piriformis test. In the lateral position, the patient pushes the heel down into the table and actively abducts with external rotation (yellow arrow) against resistance (orange arrow). The examiner palpates at the level of the piriformis.

Some specific tests are especially helpful when evaluating a patient with posterior hip pain. The seated piriformis stretch test (Fig. 2) is a passive flexion, adduction with internal rotation test performed as the examiner palpates the deep gluteal region. The active piriformis test (Fig. 3) is an active abduction and external rotation test while the examiner monitors the piriformis. The combination of the seated piriformis stretch test with the piriformis active test has shown a sensitivity of 91% and specificity of 80% for the endoscopic finding of sciatic nerve entrapment [23].

**Fig. 4.**
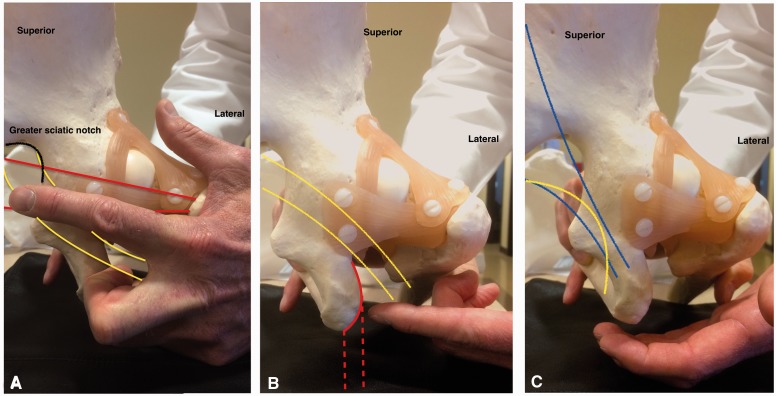
Palpation of the deep gluteal space. (A) At the greater sciatic notch (outlined in black). The path of the piriformis muscle (represented in red) and the sciatic nerve (represented in yellow). (B) Lateral to the ischium (solid red line). The path of the hamstring (dashed red line) and sciatic nerve (represented in yellow). (C) Medial to the ischium. The path of the sacrotuberous ligament (represented in blue) and the pudendal nerve (yellow line).

The palpation of the gluteal structures is fundamental for the diagnosis of gluteal pain. Using the ischial tuberosity as a reference, the production of pain by palpation can guide the probable source of symptoms as follows: at the sciatic notch, piriformis syndrome (Fig. 4A); lateral to the ischium, ischial tunnel syndrome or ischiofemoral impingement (Fig. 4B); and medial, pudendal nerve entrapment (Fig. 4C) [4].

**Fig. 5.**
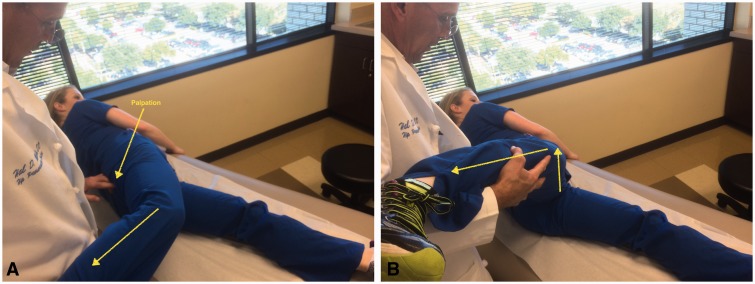
Ischiofemoral impingement test. (A) The symptomatic hip is passively taken into extension with zero abduction or adduction. A positive test is the recreation of the posterior hip pain. (B) The symptomatic hip is passively taken into extension with abduction without pain.

**Fig. 6.**
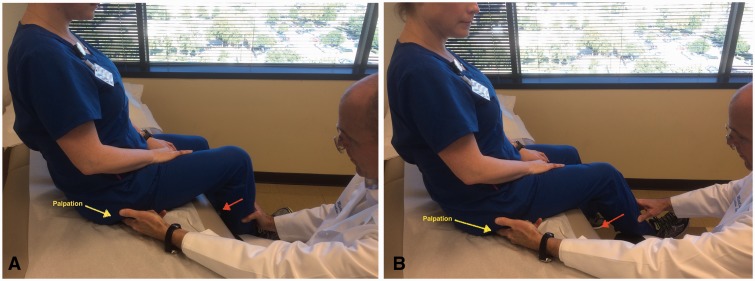
Active hamstring test. (A) The patient performs an active knee flexion against resistance with the knee at 90 degrees. Normal strength without pain may be observed. (B) The patient performs an active knee flexion against resistance with the knee at 30 degrees. Weakness and recreation of the symptoms in this position is a positive test.

The more important differential diagnoses when evaluating a patient with posterior hip pain are ischiofemoral impingement and ischial tunnel syndrome (also known as hamstring syndrome). In the first case, a provocative test should be performed with the patient in the contralateral decubitus position and taking the affected hip into passive extension. The findings of this test are considered positive when the symptoms are reproduced in adduction or the neutral position, whereas extension with abduction does not reproduce the symptoms (Fig. 5A and B). During active hamstring test muscle strength shows marked weakness and pain at 30-degree knee flexion, whereas strength is normal and pain is improved at 90-degree knee flexion (Fig. 6A and B). Differential diagnoses of deep gluteal pain are summarized in Table II.

**Table II.** Main differential diagnosis of deep gluteal syndrome

Diagnosis	History	Physical examination	Ancillary tests
Pudendal nerve entrapment	Pain in the anatomical territory of the pudendal nerve, worsened by sitting, does not wake the patient at night Numbness	Tenderness at the medial ischium	Injection guided by imaging Intrapelvic tests
Ischiofemoral impingement	Sciatic nerve complaints Lower back pain Limping	Long stride walking reproduces the pain during terminal hip extension Tenderness at the lateral ischium Positive ischiofemoral impingement test	MRI showing decreased ischiofemoral and quadratus femoris space, and quadratus femoris muscle edema.
Greater trochanter ischial impingement	Sciatic nerve complaints Laxity? Limping	Tenderness at posterior aspect of the greater trochanter Pain in deep flexion, abduction and external rotation	Injection guided by imaging
Ischial tunnel syndrome	Sciatic nerve complaints Limping Pain increased by flexion of the hip and extension of the knee	Tenderness at lateral ischium Positive hamstring active test	Injection guided by imaging MRI showing hamstring origin avulsion with edema around the sciatic nerve

### Treatment

Imaging guided intra-articular and extra-articular injections (local anesthetic and steroids) have been described to be very helpful, precise and reproducible for discriminating these complex pathologies [3]. Botulin toxin has been reported as an effective adjunt to physicial therapy in the treatment of piriformis syndrome [24], however in the experience of the senior author the botulin toxin produces more scar tissue around the sciatic nerve. The percentage of relief after injection should be investigated in order to determine how much the injected site is contributing to the pain.

In rare cases that fail conservative therapy (including physical therapy, analgesic and anti-inflammatory drugs, and injections), surgical treatment (open or endoscopic) may be considered. Several studies have reported good results after open sciatic decompression in patients with piriformis syndrome [3], hamstring syndrome (also called ischial tunnel syndrome) [6], sciatic neuropathy associated with acetabular fractures [25], post-traumatic sciatica [16], retro trochanteric pain syndrome [8] and other causes.

In 2003, Dezawa *et al*. [26] reported the endoscopic decompression of the sciatic nerve entrapment as an effective and minimally invasive approach on six cases of piriformis syndrome. Martin *et al*. [4] reported a case series of 35 patients presenting with deep gluteal syndrome treated endoscopically. Average duration of symptoms was 3.7 years with an average pre-operative verbal analog score of 7, which decreased to 2.4 post-operatively. Pre-operative modified Harris Hip Score (mHHS) was 54.4 and increased to 78 post-operatively. Twenty-one patients reported pre-operative use of narcotics for pain; 2 remained on narcotics post-operatively (unrelated to initial complaint). Eighty-three percent of patients had no post-operative sciatic sit pain (inability to sit for >30 min). Five patients experienced low mHHS scores and modest pain relief post-operatively. This poor outcome group may be related to femoral retroversion and previous abdominal surgery. Pérez-Carro *et al*. reported 26 cases of endoscopic sciatic decompression with a mean follow-up of 11 months. Nineteen cases presented excellent or good results. The mHHS went from 56 pre- to 79 post-operative on average (non-published observation, Perez-Carro L, Fernandez-Hernando M, Cerezal L: Deep gluteal pain and sciatic nerve entrapment. Presented at the ISHA Annual Scientific Meeting held in Río de Janeiro, Brazil on 9–11 October 2014).

### Endoscopic assessment and decompression technique

**Fig. 7.**
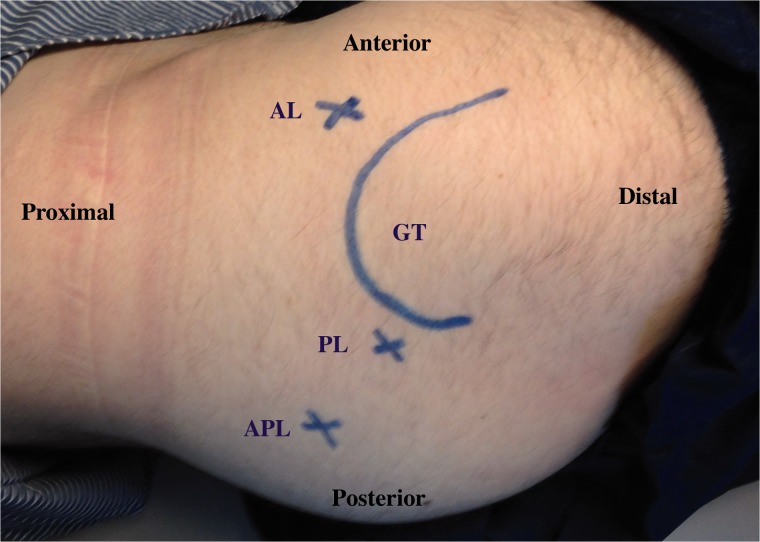
Patient in lateral position, right hip. Observe the location of the anterolateral (AL) portal, posterolateral (PL) portal and the auxiliary posterolateral (APL) portal around the greater trochanter (GT, curved line).

Endoscopy allows for a complete extrapelvic sciatic nerve visualization and safe nerve decompression in the deep gluteal space. The supine (or lateral) technique allows for manual manipulation of the lower limb at the knee and hip joints for the full assessment of sciatic nerve kinematics. A relative contra-indication for sciatic nerve decompression in this supine technique is knee recurvatum, considering the increased strain on the sciatic nerve. Nerve conduction and EMG is usually monitored intra-operatively and can demonstrate immediate improvement or change post-release. Acute variations in nerve conduction during the course of surgery may be observed due to fluid accumulation over time. For deep gluteal space visualization, a 70-degree high definition long arthroscope with adjustable and lengthening cannulas are utilized [4]. The cannulas are opened to maintain the fluid flow, when utilizing the radiofrequency probe [27]. Fluid pressure is set to 60 mmHg with intermittent pressure increases up 80 mmHg. Three portals are utilized: anterolateral, posterolateral, and an auxiliary posterolateral portal (Fig. 7). Frequent use of intra-operative fluoroscopy will confirm the proper location of the endoscopic view.

**Fig. 8.**
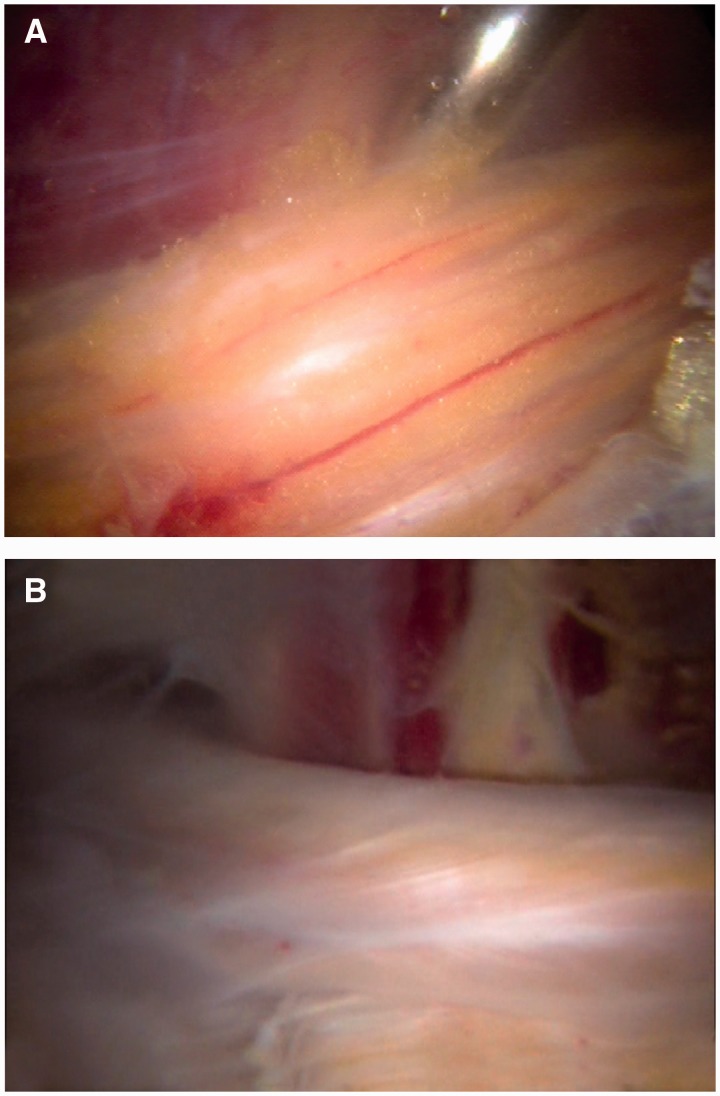
Sciatic nerve inspection. (A) Normal sciatic nerve appearance with presence of blood flow and epineural fat. (B) Abnormal sciatic nerve with white shoestring appearance, and no epineural fat.

The inspection of the sciatic nerve should include the recognition of normal versus abnormal nerve appearance and motion (Fig. 8A and B). A branch of the inferior gluteal artery must be identified and cauterized for access to the piriformis muscle. A cadaveric study has reported a mean distance of 8 mm (4–14 mm) between the sciatic nerve and the crossing branch of the inferior gluteal vessel [27]. Additionally, the temperature profile during activation of a monopolar radiofrequency device was found to be safe at a distance of 3–10 mm to the sciatic nerve during activation times of 3, 5 and 10 s [27]. The standard approach to vessel cauterization is a 3-s interval of radiofrequency activation, maintaining continuous irrigation.

A standardized technique and surgical experience for diagnosis and sciatic nerve decompression is mandatory in order to identify the sciatic nerve anatomy, avoid iatrogenic injury and to not overlook potential sources of sciatic nerve entrapment. A 10-step technique for safe sciatic nerve decompression is presented in Table III.

**Table III.** Ten recommended steps for safe sciatic nerve decompression during endoscopic technique.

To perform trochanteric bursectomy To identify the sciatic nerve and quadratus femoris muscle To evaluate the sciatic nerve To identify vascular branches around the sciatic nerve To perform endoscopic neurolysis of the sciatic nerve To perform a distal inspection To turn the scope proximally and identify the obturator internus muscle and tendon To identify the crossing branch of the piriformis muscle and tendon To identify and resect the piriformis muscle and tendon To probe the sciatic nerve for tension and look for hidden muscle or tendon branches traversing the nerve

### Post-operative rehabilitation

The rehabilitation of these complex pathologies is critical for success. The purpose of rehabilitation is to gain mobility and maintain movement of the hip joint and avoid any type of stretching of the nerve that can produce neuralgia or neuropraxy. The complete rehabilitation process takes an average of 24 weeks to return to previous activity [28].

**Fig. 9.**
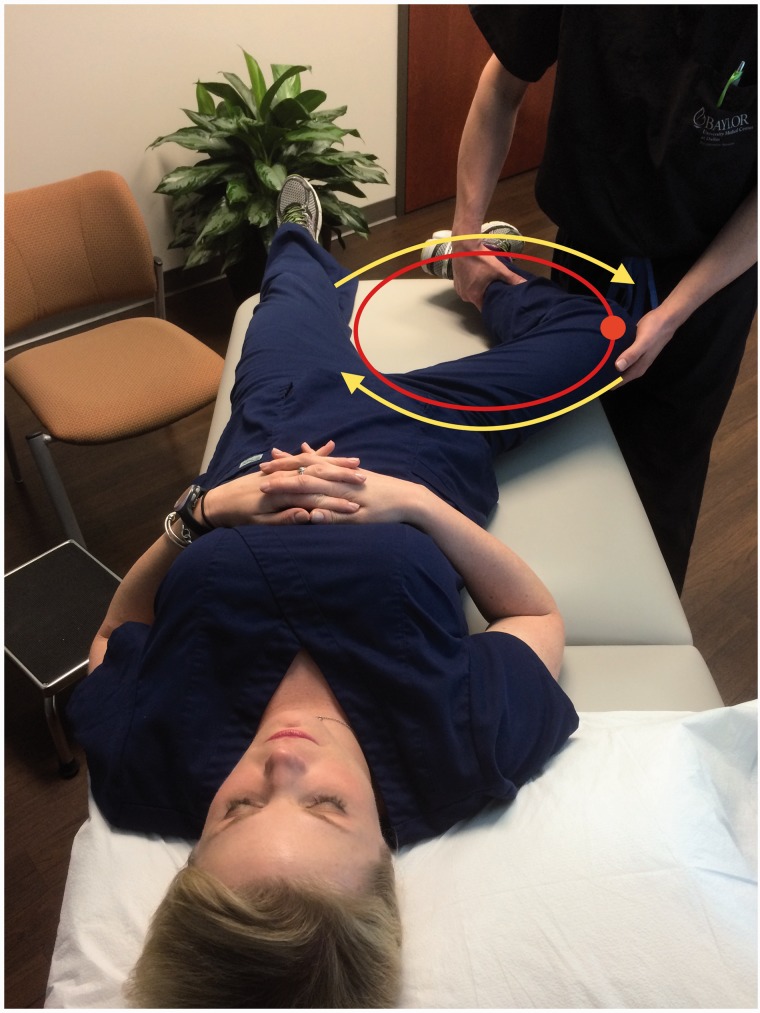
Passive hip circumductions beggining 45 degrees of hip flexion, maximum external rotation engaging the greater trochanter agains the ischium to mobilize the sciatic nerve lateral.

**Fig. 10.**
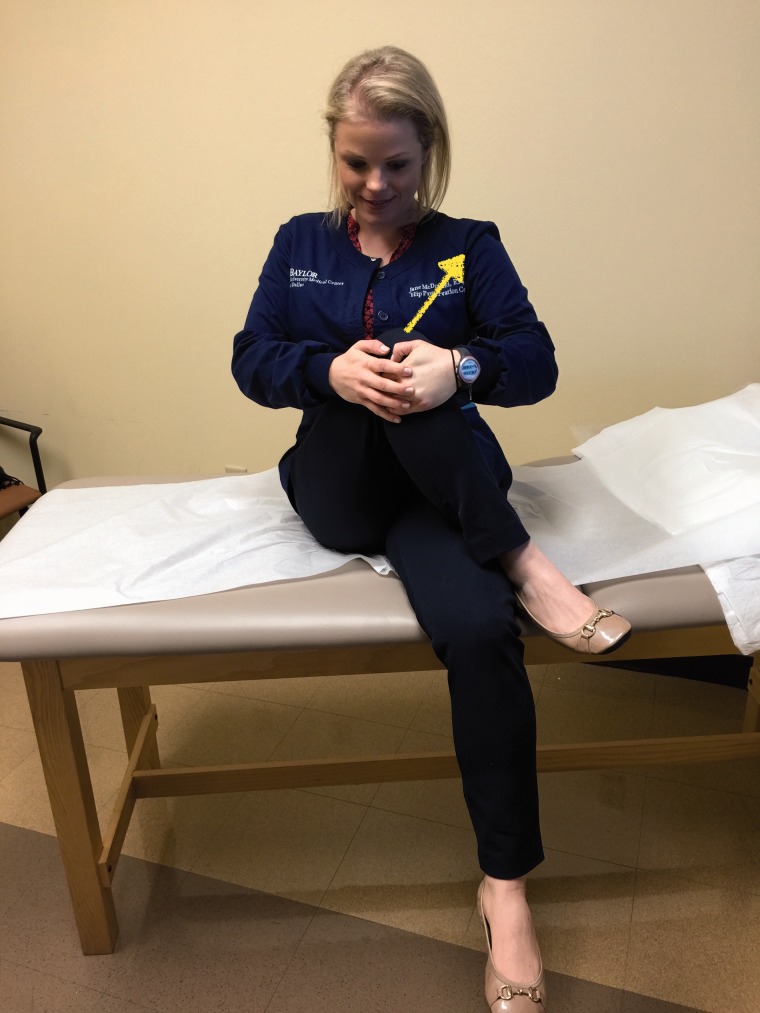
Piriformis stretch—in seated position, the patient crosses the leg that will be stretched with the foot positioned next to the knee. The stretching is performed with the patient bringing the knee towards to the contralateral shoulder. The duration and quantity of stretch is determined according with the advance of the healing process.

**Fig. 11.**
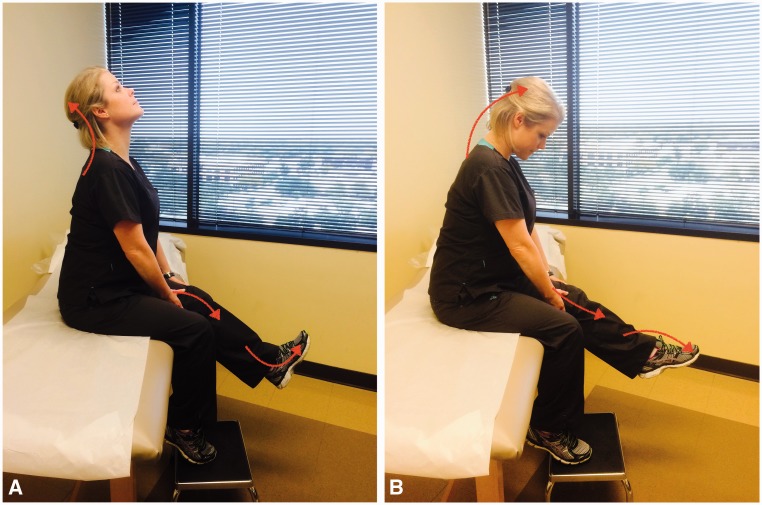
Nerve glides—patient in a siting position, hold with both hands under the knee. The exercise is performed with the mobilization of the posterior neural chain. (A) Cervical extension, knee flexion and dorsiflexion. (B) Cervical flexion, knee extension (under the limit for each phase), plantar flexion. The patient can apply lumbar flexion and extension during the exercise.

Full circumduction of the hip (engaging the greater trochanter on the ischium and pushing the sciatic nerve lateral) with knee flexion can begin on day one (Fig. 9). Piriformis stretch (Fig. 10) and nerve glides (Fig. 11A and B) can be applied under the limit of pain. A knee brace is used to avoid knee extension and maintain a relaxed sciatic nerve during therapy. The utilization of the knee brace is dependent upon the strain of the sciatic nerve, which is influenced by the degree of femoral anteversion and the number of sites of entrapment. The knee is locked at 45 degree for 3 weeks applying only nerve glides and circumduction. After 4 weeks, knee extension can increase up to 10 degree every 2 weeks as tolerated. Increase range of motion for hip flexion and adduction can be carefully applied, gentle nerve glides and include stretching maneuvers aimed at the external rotators. Standard physical therapy protocol can begin as early as 6 weeks. Again, a word of caution in cases of previous abdominal surgery and femoral retroversion as strain parameters will be a dependent factor, and the nerve may be impinged in more than one location. The therapist should be diligent in recognizing these potential outcome factors. These physical therapy techniques are the same techniques used in pre-operative conservative treatment.

The use of steroidal and non-steroidal anti-inflammatories has been found useful after day 2. Additional physical therapy techniques may be helpful including ultrasound and electrical stimulation. With a proper rehabilitation protocol, good to excellent outcomes can be achieved. The advancement in rehabilitation has proven beneficial to improve the outcomes of endoscopic sciatic nerve decompression.

At the present time, among 200 international cases have addressed extrapelvic deep gluteal syndrome and complications continue to be extremely low. To help avoid post-surgical stretch injury, it is recommended that intra-articular work be performed separately from extra-articular work. Due to the length of time from diagnosis to treatment and recovery, the psychological toll of the pain cycle can be frustrating. Complications have involved hematomas brought on by early post-operative use of NSAIDs with excessive post-operative activity. Concomitant pudendal nerve and sciatic nerve complaints are often resolved, however in two of those 200 cases the pudendal complaints worsened.

## CONCLUSION

The understanding of deep gluteal syndrome continues to evolve with the recognition of the various sites that the sciatic nerve can be entrapped. A comprehensive history and physical examination with specialized testing including injections will aid in the differential diagnosis of posterior hip pain. Open or endoscopic decompression of the sciatic nerve in cases that fail conservative therapy are useful in improving function and diminishing pain.

## CONFLICT OF INTEREST STATEMENT

None declared.

## References

[1] McCrory P Bell S Nerve entrapment syndromes as a cause of pain in the hip, groin and buttock. Sports Med 1999; 27: 261–74. 10367335 10.2165/00007256-199927040-00005

[2] Robinson DR Pyriformis syndrome in relation to sciatic pain. Am J Surg 1947; 73: 355–8. 20289074 10.1016/0002-9610(47)90345-0

[3] Filler AG Haynes J Jordan SE Sciatica of nondisc origin and piriformis syndrome: diagnosis by magnetic resonance neurography and interventional magnetic resonance imaging with outcome study of resulting treatment. J Neurosurg Spine 2005; 2: 99–115. 15739520 10.3171/spi.2005.2.2.0099

[4] Martin HD Shears SA Johnson JC The endoscopic treatment of sciatic nerve entrapment/deep gluteal syndrome. Arthroscopy 2011; 27: 172–81. 21071168 10.1016/j.arthro.2010.07.008

[5] Puranen J Orava S The hamstring syndrome: a new diagnosis of gluteal sciatic pain. Am J Sports Med 1988; 16: 517–21. 3189686 10.1177/036354658801600515

[6] Young IJ van Riet RP Bell SN Surgical release for proximal hamstring syndrome. Am J Sports Med 2008; 36: 2372–8. 18818432 10.1177/0363546508322905

[7] Cox JM Bakkum BW Possible generators of retrotrochanteric gluteal and thigh pain: the gemelli-obturator internus complex. J Manipulative Physiol Ther 2005; 28: 534–8. 16182029 10.1016/j.jmpt.2005.07.012

[8] Meknas K Kartus J Letto JI Surgical release of the internal obturator tendon for the treatment of retro-trochanteric pain syndrome: a prospective randomized study, with long-term follow-up. Knee Surg Sport Traumatol Arthrosc 2009; 17: 1249–56. 10.1007/s00167-009-0787-z 19396428

[9] Labropoulos N Tassiopoulos AK Gasparis AP Veins along the course of the sciatic nerve. J Vasc Surg 2009; 49: 690–6. 19135832 10.1016/j.jvs.2008.09.061

[10] Papadopoulos SM McGillicuddy JE Albers JW Unusual cause of “piriformis muscle syndrome”. Arch Neurol 1990; 47: 1144–6. 2222250 10.1001/archneur.1990.00530100114027

[11] Beauchesne RP Schutzer SF Myositis ossificans of the piriformis muscle: an unusual cause of piriformis syndrome. J Bone Joint Surg Am 1997; 79: 906–10. 9199390 10.2106/00004623-199706000-00016

[12] Coppieters MW Alshami AM Babri AS Strain and excursion of the sciatic, tibial, and plantar nerves during a modified straight leg raising test. J Orthop Res 2006; 24: 1883–9. 16838375 10.1002/jor.20210

[13] Smoll NR Variations of the piriformis and sciatic nerve with clinical consequence: a review. Clin Anat 2010; 23: 8–17. 19998490 10.1002/ca.20893

[14] Wall E Massie J Experimental stretch neuropathy. Changes in nerve conduction under tension. J Bone Joint Surg Br 1992; 74: 126–9. 1732240 10.1302/0301-620X.74B1.1732240

[15] Martin R Kivlan B Martin HD Greater Trochanter-Ischial Impingement: A Potential Source of Posterior Hip Pain. Rio de Janeiro: International Society for Hip Arthroscopy, 2014.

[16] Benson B Schutzer SF Posttraumatic Piriformis syndrome: diagnosis and results of operative treatment. J Bone Joint Surg Am 1999; 81: 941–9. 10428125

[17] Papadopoulos EC Khan SN Piriformis syndrome and low back pain: a new classification and review of the literature. Orthop Clin North Am 2004; 35: 65–71. 15062719 10.1016/S0030-5898(03)00105-6

[18] Blankenbaker DG Tuite MJ Non—femoroacetabular impingement. Semin Musculoskelet Radiol 2013; 17: 279–85. 23787982 10.1055/s-0033-1348094

[19] Possover M Laparoscopic management of endopelvic etiologies of pudendal pain in 134 consecutive patients. J Urol 2009; 181: 1732–6. 19233408 10.1016/j.juro.2008.11.096

[20] Rivera J Singh V Fellows B Reliability of psychological evaluation in chronic pain in an interventional pain management setting. Pain Phys 2005; 8: 375–83. 16850061

[21] Martin HD Palmer IJ History and physical examination of the hip: the basics. Curr Rev Musculoskelet Med 2013; 6: 219–25. 23832778 10.1007/s12178-013-9175-x PMC4094013

[22] Martin HD Kelly BT Leunig M The pattern and technique in the clinical evaluation of the adult hip: the common physical examination tests of hip specialists. Arthroscopy 2010; 26: 161–72. 20141979 10.1016/j.arthro.2009.07.015

[23] Martin HD Kivlan BR Palmer IJ Diagnostic accuracy of clinical tests for sciatic nerve entrapment in the gluteal region. Knee Surg Sports Traumatol Arthrosc 2014; 22: 882–8. 24217716 10.1007/s00167-013-2758-7

[24] Fishman LM Anderson C Rosner B BOTOX and physical therapy in the treatment of piriformis syndrome. Am J Phys Med Rehabil 2002; 81: 936–42. 12447093 10.1097/00002060-200212000-00009

[25] Issack PS Kreshak J Klinger CE Sciatic nerve release following fracture or reconstructive surgery of the acetabulum. Surgical technique. J Bone Joint Surg Am 2008; 90(Suppl 2): 227–37. 18829936 10.2106/JBJS.H.00120

[26] Dezawa A Kusano S Miki H Arthroscopic release of the piriformis muscle under local anesthesia for piriformis syndrome. Arthroscopy 2003; 19: 554–7. 12724687 10.1053/jars.2003.50158

[27] Martin HD Palmer IJ Hatem M Monopolar radiofrequency use in deep gluteal space endoscopy: sciatic nerve safety and fluid temperature. Arthroscopy 2014; 30: 60–4. 24183195 10.1016/j.arthro.2013.08.034

[28] Martin HD Subgluteal space and associated disorders. In: Byrd T (ed.). Operative Hip Arthroscopy. Germany: Springer, 2013, 309–29.

